# Pantoprazole-induced anaphylactic asthma and Type I Kounis syndrome: a case report and literature review

**DOI:** 10.3389/fcvm.2026.1883834

**Published:** 2026-09-29

**Authors:** Ailing Huang, Songyun Li, Haseeb Sattar, Yalu Qin, Kaixin Tian, Qian Nie

**Affiliations:** 1 Department of Cardiology, Chengdu Pidu District Hospital of Traditional Chinese Medicine, Chengdu, China; 2 Department of Cardiology, Affiliated Hospital of Chengdu University of Traditional Chinese Medicine, Chengdu, Sichuan, China; 3 Department of Medicine, Urban Vocational College of Sichuan, Chengdu, China

**Keywords:** anaphylactic asthma, anaphylaxis, Kounis syndrome, myocardial bridging, pantoprazole

## Abstract

**Background:**

Proton pump inhibitors (PPIs) are among the most frequently prescribed medications and are generally considered to have a favourable safety profile. Nevertheless, immediate hypersensitivity reactions may occur and, in exceptional circumstances, can precipitate Kounis syndrome (KS), an acute coronary syndrome resulting from the activation of inflammatory pathways. We describe an elderly patient who developed severe anaphylaxis with Type I KS shortly after intravenous pantoprazole administration in the presence of an unsuspected myocardial bridge, and discuss the potential clinical implications.

**Case summary:**

An 85-year-old woman with a 30-year history of well-controlled bronchial asthma was admitted with community-acquired pneumonia. During hospitalization, she received intravenous pantoprazole (40 mg) for acid reflux. Within five minutes of the infusion, she developed a widespread erythematous rash, severe bronchospasm, profound hypotension (74/56 mmHg), and ST-segment elevation of approximately 0.3 mV in leads I, aVL, and V₁ to V₄. Emergency coronary angiography demonstrated no obstructive coronary artery disease but identified a myocardial bridge involving the mid-left anterior descending artery, producing nearly 90% systolic compression. Serial cardiac troponin and NT-proBNP concentrations remained within the normal range. Assessment using the Naranjo Adverse Drug Reaction Probability Scale yielded a score of six, indicating a probable causal relationship between pantoprazole and the adverse event. The patient was treated with methylprednisolone (80 mg). The recovery occurred within 2 h.

**Conclusion:**

This case highlights that intravenous pantoprazole may rarely trigger life-threatening Type I Kounis syndrome. It also raises the possibility that a pre-existing myocardial bridge can accentuate coronary vasospasm during severe allergic reactions, thereby contributing to the clinical presentation. Based on this experience and the available literature, we propose a practical management algorithm to facilitate timely diagnosis and treatment in similar clinical settings.

## Introduction

Pantoprazole, a substituted benzimidazole proton pump inhibitor (PPI), exerts potent gastric acid suppression through irreversible inhibition of the H⁺/K⁺-ATPase in gastric parietal cells. It is among the most prescribed medications worldwide for the management of gastroesophageal reflux disease, peptic ulcer disease, and stress ulcer prophylaxis (1). Although its safety profile is generally favorable, with most adverse effects limited to mild gastrointestinal or neurological complaints, hypersensitivity reactions — ranging from benign urticaria to life-threatening anaphylaxis have been sporadically reported (2, 3).

Kounis syndrome (KS), first conceptualized by Kounis and Zavras in 1991, describes the coincident occurrence of an acute coronary syndrome with an allergic, hypersensitivity, or anaphylactic reaction (4). The syndrome is currently classified into four types: type I features transient coronary spasm in the absence of significant pre-existing coronary artery disease as shown in Figure 1, Type II (plaque erosion or rupture in patients with pre-existing atheromatous disease), Type III (stent thrombosis in patients with drug-eluting or bare-metal stents), and the recently proposed Type IV (coronary artery bypass graft thrombosis) (5–7). The underlying pathophysiology centres on the degranulation of mast cells and other inflammatory cells, with the release of potent vasoactive mediators — histamine, leukotrienes, platelet-activating factor, and tryptase that can provoke intense coronary vasoconstriction, promote plaque destabilization, or trigger stent thrombosis (8).

**Figure 1:**
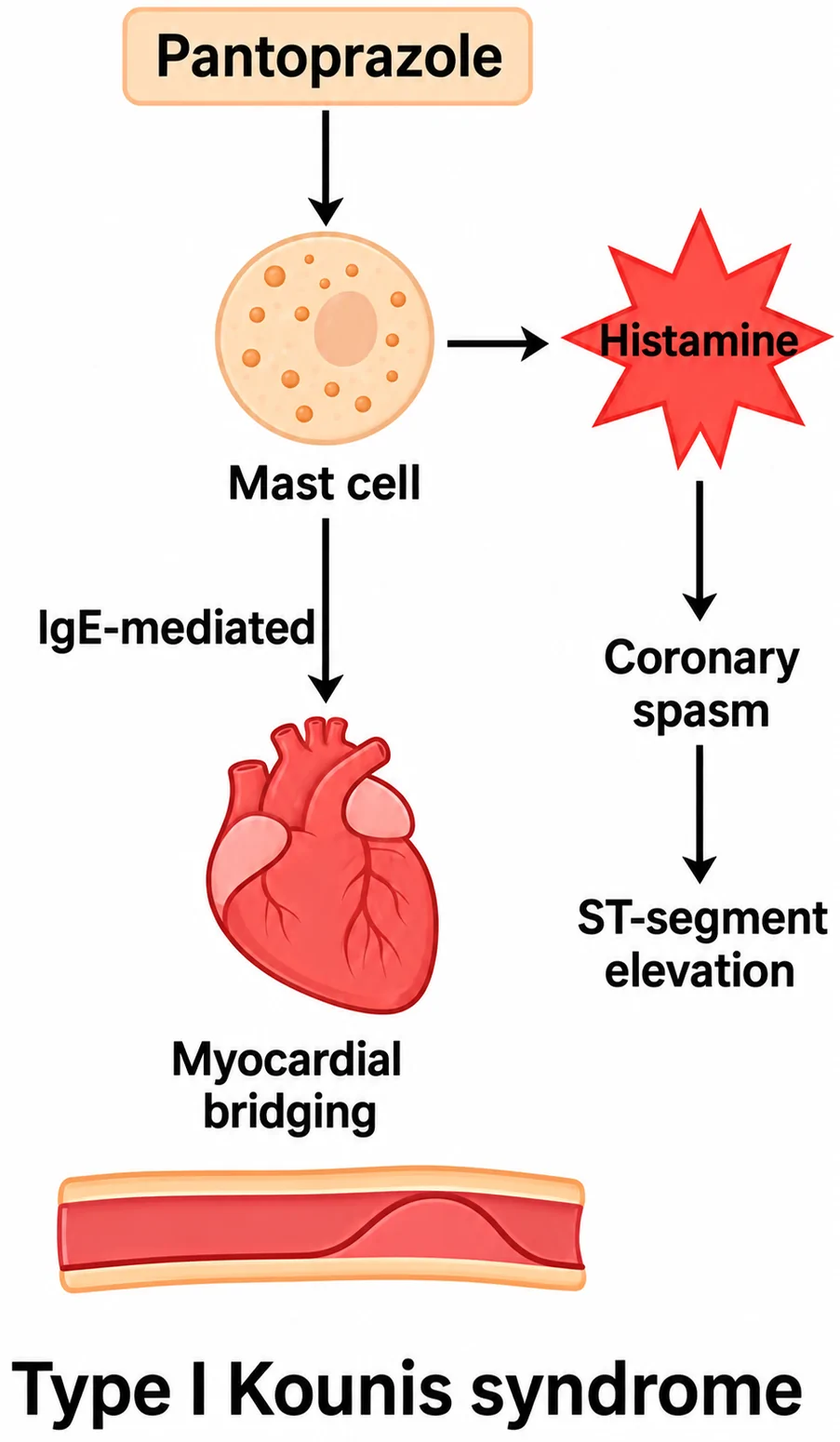
Mechanism of type I Kounis syndrome.

The intersection of PPI-induced anaphylaxis and Kounis syndrome is exceedingly rare. To date, only three specific PPIs have been implicated in published case reports: pantoprazole (9), omeprazole (10), and lansoprazole (11). The index case of pantoprazole-induced KS was reported by Canpolat et al. in 2012 (9), describing a 76-year-old man who developed acute coronary syndrome following oral pantoprazole. Subsequently, Ghilencea et al. in 2020 reported Type I KS in association with myocardial bridging after protracted anaphylaxis from a non-PPI trigger (12), and an earlier 2011 case described the combination of Type I KS and myocardial bridging leading to myocardial infarction in a young patient after clindamycin (13). However, the concurrent occurrence of pantoprazole-induced anaphylaxis, acute bronchospasm, and Type I KS in the setting of a severe myocardial bridge has not been previously reported in an elderly patient. We present this case to: (1) highlight the potential for commonly prescribed PPIs to trigger catastrophic allergic coronary events; (2) demonstrate how underlying anatomical variants such as myocardial bridges can dramatically amplify ischemic sequelae; (3) provide a structured diagnostic and therapeutic algorithm for clinicians encountering suspected drug-induced KS; and (4) discuss implications for medication safety in elderly, multimorbid populations.

## Case presentation

### Patient information and medical history

An 85-year-old Chinese woman presented to the emergency department with a 3-day history of productive cough following an upper respiratory tract infection. She had a 30-year history of bronchial asthma that was well-controlled with inhaled budesonide/formoterol (160/4.5 µg twice daily). She denied any known drug allergies, including prior exposure to PPIs. She had no history of coronary artery disease, hypertension, diabetes mellitus, or smoking. Family history was notable for myocardial infarction in her father at age 72; there was no family history of allergic or anaphylactic disorders. The patient lived independently and had no prior hospitalizations for cardiac or allergic conditions.

### Clinical findings on admission

On admission, vital signs were: blood pressure 128/72 mmHg, heart rate 76 beats/min, respiratory rate 18 breaths/min, temperature 38.1 °C, and oxygen saturation 95% on room air. Chest auscultation revealed coarse breath sounds bilaterally without wheezing or crackles. Cardiac examination was unremarkable; no murmurs, gallops, or rubs were appreciated. Laboratory investigations showed: white blood cell count 21.57 × 10⁹/L (reference 3.50–9.50), neutrophil percentage 85.6%, eosinophil count 0 × 10⁹/L (reference 0.02–0.52), high-sensitivity C-reactive protein 8.86 mg/L (reference < 5.00), and procalcitonin 6.72 ng/mL (reference 0–0.08). Sputum Gram stain revealed Gram-positive cocci (2+) and Gram-negative bacilli (4+). Chest computed tomography demonstrated minor chronic inflammatory changes in the bilateral lower lobes and a small bulla in the right lower lobe. Cardiac and abdominal colour Doppler ultrasound were unremarkable. The admission diagnosis was community-acquired pneumonia.

### Therapeutic intervention and adverse event

The patient was initiated on intravenous moxifloxacin (400 mg daily) for anti-infective therapy. After three days, her cough improved substantially (resolved to occasional dry cough), and she remained afebrile. However, she developed acid reflux and poor appetite, for which intravenous pantoprazole (40 mg in 100 mL normal saline, infused over 15 min) was prescribed.

Approximately 5 min after the infusion began, the patient abruptly developed a generalized, intensely pruritic erythematous rash over the trunk and both upper arms (Figures 2a,b). This was rapidly followed by pallor, perioral cyanosis, severe dyspnea with audible wheezing, palpitations, and substernal chest pain (numeric rating scale 7/10, described as “pressure-like”). Bedside monitoring revealed: blood pressure 74/56 mmHg, heart rate 82 beats/min, oxygen saturation 88% on room air, and diffuse expiratory wheezing throughout both lung fields. An immediate 12-lead electrocardiogram (ECG) demonstrated ST-segment elevation of 0.3 mV with upward convexity in leads I, aVL, and V₁–V₄ (Figure 2C). Bedside transthoracic echocardiography revealed hypokinesia of the anterior interventricular septum with preserved global left ventricular systolic function and no structural abnormalities. Arterial blood gas analysis confirmed hypoxemia (PaO₂ 62 mmHg on room air). Notably, serial high-sensitivity cardiac troponin I and NT-proBNP levels remained within normal limits throughout the acute episode.

**Figure 2:**
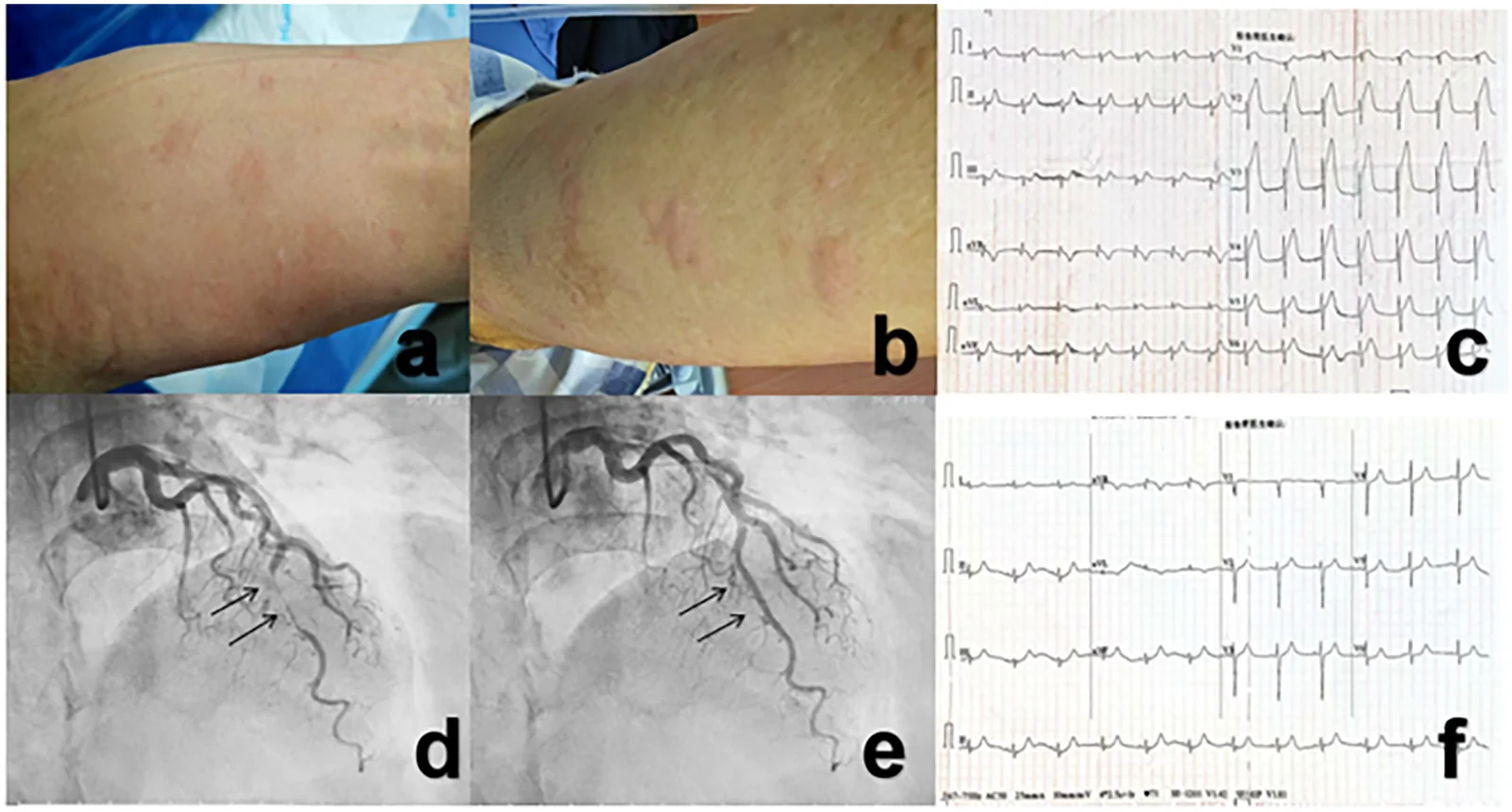
Clinical images. Panel **(a,b)** Erythematous rash on both upper arms; panel **(c)** Admission ECG demonstrating ST-segment elevation (0.3 mV) in leads I, aVL, and V₁–V₄; **(d,e)** Coronary angiography in systole panel **(d)** and diastole panel **(e)** showing myocardial bridge in the mid-LAD with approximately 90% systolic compression (arrow); panel **(f)** Follow-up ECG after symptom resolution demonstrating complete ST-segment normalization.

### Diagnostic assessment and clinical reasoning

The differential diagnosis was investigated, including: (a) Type I Kounis syndrome secondary to pantoprazole-induced anaphylaxis; (b) acute ST-segment elevation myocardial infarction (STEMI) due to plaque rupture; (c) stress (Takotsubo) cardiomyopathy; (d) acute pulmonary embolism; and (e) acute pericarditis. The close temporal relationship (≤5 min after IV pantoprazole), concomitant cutaneous and respiratory manifestations consistent with anaphylaxis, and the complete absence of fixed coronary stenosis on angiography strongly supported Type I KS rather than conventional STEMI. The absence of regional wall motion abnormalities extending beyond a single coronary territory, together with normal troponin, excluded Takotsubo cardiomyopathy. Pulmonary embolism was considered unlikely based on the overall clinical findings and normal D-dimer. The convex upward ST morphology on ECG argued against acute pericarditis.

### Naranjo adverse drug reaction probability assessment

The Naranjo Adverse Drug Reaction Probability Scale (14) was calculated, i.e., 6 (probable causality). The scoring breakdown was as follows: (1) previous reports of reaction (+1); (2) adverse event occurred after the suspected drug administration (+2); (3) patient condition improved upon drug discontinuation (+1); (4) no alternative causes identified (+2); (5) no placebo or rechallenge performed (0); (6) drug not detected in body fluids (0); (7) no previous history of similar reaction(0); (8) objective evidence of the adverse event confirmed by ECG and angiography (+1); (9) no placebo or rechallenge (−1 for absence of rechallenge). Total=6.

### Therapeutic intervention and clinical course

Pantoprazole infusion was stopped immediately once the clinical picture became consistent with an acute anaphylactic reaction accompanied by myocardial ischaemia. Initial management focused on stabilising the patient's airway and circulation. High-flow oxygen was delivered through a non-rebreather mask at 10 L/min, followed by I/V methylprednisolone (80 mg) and diphenhydramine (20 mg). To treat the hypotension, normal saline was given in divided doses with a total volume of 500 mL.

The patient's condition improved rapidly after treatment. Cutaneous manifestations began to resolve within approximately 30 min, bronchospasm became substantially less pronounced, and blood pressure increased from 74/56 mmHg to 102/68 mmHg. Chest discomfort settled completely over the subsequent 90 min, accompanied by progressive haemodynamic stabilisation.

Although intramuscular epinephrine remains the cornerstone of treatment for severe anaphylaxis, it was intentionally withheld in this patient because the combination of acute allergic manifestations and ST-segment elevation raised a strong suspicion of Type I Kounis syndrome. In our case, epinephrine may theoretically aggravate coronary vasospasm through its *α*₁-adrenergic vasoconstrictor effects and potentially worsen myocardial ischaemia. For this reason, the initial therapeutic strategy emphasised corticosteroids, antihistamines, oxygen supplementation, and intravenous fluids. Epinephrine was reserved as a rescue option, because it should be used cautiously when Kounis syndrome is suspected (15–17).

Despite the patient's early clinical improvement, persistent ST-segment elevation warranted urgent coronary angiography to exclude an acute occlusive coronary event. The procedure, performed approximately three hours after symptom onset, demonstrated angiographically normal coronary arteries without evidence of obstructive atherosclerotic disease. An incidental myocardial bridge was identified in the mid-segment of the left anterior descending artery, producing approximately 90% systolic luminal compression with complete restoration of vessel calibre during diastole, consistent with the characteristic milking phenomenon (Figures 2d,e). Provocative testing with ergonovine or acetylcholine was not undertaken because the temporal relationship with pantoprazole exposure was compelling, no fixed coronary stenosis was present, and deliberate re-induction of coronary spasm in a patient who had recently experienced severe anaphylaxis was considered neither necessary nor ethically appropriate.

A repeat electrocardiogram obtained approximately 4 h after symptom onset showed complete resolution of the previously observed ST-segment elevation (Figure 2F). Serial measurements of cardiac troponin I at six and twelve hours remained below the assay detection threshold (<0.01 ng/mL), while follow-up transthoracic echocardiography demonstrated recovery of the transient septal wall-motion abnormality. Pantoprazole was discontinued permanently, and gastric acid suppression was maintained with famotidine 20 mg twice daily. No adverse reactions occurred following the change in therapy, and the remainder of the patient's hospital stay was uneventful (Table 1).

**Table 1:** presents a structured clinical timeline summarizing the key events from admission through discharge.

Time	Phase	Event
Day 1	Admission	Cough×3d; WBC 21.57; PCT 6.72; CT: mild chronic inflammation; Diagnosed CAP; IV moxifloxacin started
Day 1–3	Treatment	Cough significantly improved; afebrile; acid reflux developed
Day 3, 14:00	Trigger	IV pantoprazole 40 mg infusion started
Day 3, 14:05	Anaphylaxis onset	Rash, bronchospasm, BP 74/56, SpO₂ 88%, chest pain, ST↑ I/aVL/V₁_-_₄
Day 3, 14:05–14:30	Emergency Rx	Pantoprazole stopped; O₂ 10 L/min; IV methylprednisolone 80 mg; IV diphenhydramine 20 mg; IVF
Day 3, 14:35	Response	Rash fading; wheezing ↓; BP 102/68; chest pain persists but improving
Day 3, 17:00	Coronary angio	LAD myocardial bridge (90% systolic compression); no fixed stenosis → Type I KS confirmed
Day 3, 18:00	ECG normalized	ST-segment normalization; troponin <0.01; chest pain fully resolved
Day 3–10	Inpatient obs	Serial troponins negative; famotidine started; PPI allergy documented; patient education
Week 4 & 12	Follow-up	Outpatient cardiology FU: asymptomatic; ECG normal; stress echo normal; PPI allergy alert card issued

## Discussion

The co-occurrence of myocardial bridging and KS was first documented in a 17-year-old who developed an acute anterolateral myocardial infarction following clindamycin administration (13), and subsequently in an adult case after protracted non-drug anaphylaxis (12). These reports, together with our case, suggest that myocardial bridges should be recognized as anatomical “hot spots” of susceptibility during systemic allergic reactions. The intramyocardial segment, already subject to systolic compression under basal conditions, may become exquisitely sensitive to the vasospastic effects of histamine (via H1 receptor activation) and cysteinyl leukotrienes (via direct smooth muscle contraction and endothelial barrier disruption) released during mast cell degranulation (18, 19).

### Pathophysiological mechanisms of PPI-induced Kounis syndrome

The antigenic basis of PPI hypersensitivity is attributed to the pyridine and sulfinyl functional groups common to all substituted benzimidazole PPIs (20). These haptens covalently bind to host carrier proteins, forming antigens that can elicit specific IgE production in genetically susceptible individuals (21). Upon re-exposure, antigen-specific IgE bound to high-affinity Fc*ε*RI receptors on mast cells and basophils becomes cross-linked, triggering immediate degranulation with release of preformed mediators (histamine, tryptase, chymase, TNF-α) and *de novo* synthesis of arachidonic acid metabolites (cysteinyl leukotrienes LTC₄, LTD₄, LTE₄; prostaglandin D₂; platelet-activating factor) (22, 23). The literature on PPI hypersensitivity (21) emphasises that diagnosis should be confirmed through a combination of clinical history, skin testing, and drug provocation testing, where feasible. However, cross-reactivity among PPIs is highly variable, ranging from 0% to 68% depending on the specific agents tested, making it essential to test all available PPIs before labelling a patient as “allergic to all PPIs” (24).

Notably, the patient's admission eosinophil count was 0 × 10⁹/L. While peripheral eosinophilia is classically associated with allergic disorders, severe anaphylaxis is often accompanied by transient eosinopenia due to marginalization of circulating eosinophils and tissue sequestration of eosinophils (25). Concurrent acute bacterial pneumonia (procalcitonin 6.72 ng/mL) further suppressed eosinophil counts, likely through infection-induced cortisol release and cytokine-mediated apoptosis. This finding indicates the limited diagnostic utility of peripheral eosinophil counts in the acute setting of suspected drug-induced anaphylaxis and highlights the need for more specific biomarkers (such as serum tryptase).

### Proposed management process

For suspected drug-induced Kounis syndrome (KS), the following dual-pathway management framework is adopted (Table 2). This process emphasizes the simultaneous management of the allergic cascade and the acute coronary event, while fully accounting for the unique pharmacological factors associated with KS.

**Table 2:** Proposed dual-pathway management algorithm for suspected drug-induced Kounis syndrome.

Targeting pathways	Recommended intervention	Rationale/cautions
1. Immediate actions	Stop suspected drug; secure airway; high-flow O₂; continuous ECG & vital sign monitoring	Remove ongoing antigenic stimulus; prevent hypoxemia
2. Allergic pathway	IV methylprednisolone 1–2 mg/kg OR hydrocortisone 200 mg; IV diphenhydramine 25–50 mg; IV fluids (crystalloid bolus)	Epinephrine preferred as first-line in KS; antihistamines block H1-mediated vasospasm
3. Coronary pathway	Nitroglycerin SL/IV for vasospasm; calcium channel blocker (diltiazem or verapamil) for persistent spasm; avoid beta-blockers (unopposed alpha may worsen spasm)	CCBs are spasmolytic; beta-blockers relatively contraindicated in vasospastic angina
4. Epinephrine decision	WITHHOLD as first-line in KS. Reserve for refractory hypotension despite fluids. If necessary: 0.1–0.3 mg IM (NOT IV bolus), with continuous ECG	Alpha-adrenergic agonism may paradoxically worsen coronary spasm and extend infarct size in KS (15, 16)
5. Diagnostic workup	Serial 12-lead ECG; serial hs-cTnI; bedside TTE; urgent coronary angiography to classify KS type	Angiography required to distinguish Type I (normal/subcritical) from Type II (plaque rupture); OCT/IVUS if available to assess plaque and wall structure
6. Biomarkers	Serum tryptase at 1–2 h, 24 h, and baseline (≥2 wks later); N-methylhistamine (24 h urine); specific IgE to suspected drug; consider PGD2	Tryptase is the gold-standard biomarker for mast cell degranulation; serial measurements improve sensitivity (26, 27)
7. Post-acute care	Cardiac monitoring×24–72 h; allergy/immunology consultation; document drug allergy; issue medical alert identification; consider H2RA as alternative gastric protection	Cross-reactivity among PPIs is unpredictable; H2RAs are structurally distinct and generally safe alternatives

CCB, calcium channel blocker; ECG, electrocardiogram; H2RA, histamine-2 receptor antagonist; hs-cTnI, high-sensitivity cardiac troponin I; IM, intramuscular; KS, Kounis syndrome; OCT, optical coherence tomography; IVUS, intravascular ultrasound; PGD2, prostaglandin D2; SL, sublingual; TTE, transthoracic echocardiography.

### Limitations

This case report has several limitations that should be acknowledged. First, serum tryptase—the gold standard biomarker for mast cell degranulation (26)—was not measured during the acute episode or at baseline. As suggested by Yale et al. (27), serial tryptase measurements (1–2 h post-reaction, 24 h, and a baseline value ≥2 weeks later) substantially increase the sensitivity for confirming anaphylaxis, and their absence in this case limits the biochemical certainty of the diagnosis. N-methylhistamine (24 h urine) and prostaglandin D₂ levels were also not obtained. Second, intravascular imaging modalities such as optical coherence tomography (OCT) and intravascular ultrasound (IVUS) were not performed during coronary angiography. While coronary angiography excluded fixed obstructive stenosis, OCT/IVUS would have provided additional information about vessel wall structure, potential plaque erosion, and intramural hematoma—findings that could further refine KS subtype classification. Third, provocative vasospasm testing (ergonovine or acetylcholine) was not performed due to the clear temporal relationship with drug exposure and ethical concerns regarding provocation in a patient with documented severe anaphylaxis. Fourth, skin prick testing or basophil activation testing for pantoprazole and cross-reactive PPIs were deferred to the outpatient setting and had not been completed at the time of this report. Fifth, the generalizability of this single case is inherently limited. The proposed management algorithm, while based on the best available evidence, has not been validated in prospective studies. Despite these limitations, the contrasting lines of clinical evidence—temporal association, multisystem anaphylactic manifestations, characteristic ECG changes, angiographic confirmation of type I KS, and complete resolution upon drug withdrawal—provide a strong clinical diagnosis according to established criteria.

## Conclusion

In conclusion, pantoprazole rarely triggers life-threatening anaphylaxis and Type I Kounis syndrome. High clinical suspicion is required, especially if multiple organ systems are involved, and the cardiac symptoms occur rapidly after drug administration. Cardiac anomalies such as myocardial bridges can significantly worsen ischemia. This case highlights that such anatomical variants can act as potent amplifiers of allergic cardiac manifestations. Early diagnosis, prompt discontinuation of the offending drug and management of both allergic and coronary ischemia are critical for a successful outcome.

## Data Availability

The raw data supporting the conclusions of this article will be made available by the authors, without undue reservation.
